# Patient Factors Associated With Vision Complaints in Idiopathic Intracranial Hypertension: A Cross-Sectional Study

**DOI:** 10.7759/cureus.90830

**Published:** 2025-08-23

**Authors:** Leah O Grcevich, Maxwell J Jabaay, Stephen L Aita, Vincent S Alexander, Michael Ernst, Hassan N Kesserwani

**Affiliations:** 1 Department of Research, Alabama College of Osteopathic Medicine, Dothan, USA; 2 Department of Research, Mayo Clinic, Rochester, USA; 3 Department of Psychiatry, Geisel School of Medicine at Dartmouth College, Hanover, USA; 4 Department of Neurology, Flowers Medical Group, Dothan, USA

**Keywords:** idiopathic intracranial hypertension, intracranial pressure, lumbar puncture, pseudotumor cerebri, ptc syndrome, translaminar pressure difference

## Abstract

Introduction

Idiopathic intracranial hypertension (IIH), formerly known as pseudotumor cerebri, is characterized by elevated intracranial pressure (ICP) and papilledema in the absence of focal neurological deficits or structural brain abnormalities. This study aimed to describe the demographic and clinical characteristics of a sample of patients with IIH and examine associations between visual complaints and various demographic, clinical, and ophthalmologic variables.

Methods

This retrospective cross-sectional study included 106 patients diagnosed with IIH in a neurology practice in the Southeast United States. Demographic data, clinical features, and ophthalmologic parameters were collected, including intraocular pressure (IOP) measurements and reports of visual complaints (blurred vision, vision loss, and transient visual obscuration). Lumbar punctures were conducted in either a seated (n=46) or lateral decubitus (n=60) position. Translaminar pressure difference (TLPD) was calculated. Chi-squared tests were used for categorical comparisons, and t-tests were used for continuous variables. Primary outcomes included the presence of visual complaints and their relationship with ICP, IOP, TLPD, and other clinical factors.

Results

Overall, 61% of patients reported at least one form of visual complaint, with symptom-specific prevalence ranging from 12% to 37%. In the lateral decubitus group, blurred vision occurred with higher TLPD (mean 44.0 vs 34.3, p=0.03, d=0.82). In the lateral decubitus group, blurred vision occurred with higher TLPD (mean 44.0 vs 34.3, p=0.03, Cohen's d=0.82). Vision loss occurred with lower IOP (mean 26.3 vs 36.1 mmHg, p=0.04, Cohen's d=1.09). Any vision complaint was associated with higher ICP (mean 38.0 vs 34.6 mmHg, p=0.04, Cohen's d=0.44).

Conclusions

IOP and TLPD showed outcome-specific associations with visual complaints, particularly blurred vision in patients sampled in lateral decubitus. These pressure measures may help risk-stratify visual symptoms in IIH and potentially warrant prospective validation.

## Introduction

Idiopathic intracranial hypertension (IIH), formerly known as pseudotumor cerebri, is characterized by increased intracranial pressure (ICP), leading to optic disc edema in the absence of a cerebral space-occupying lesion, ventriculomegaly, or an inflammatory cerebrospinal fluid (CSF) [1]. PTC nomenclature usually has a proximate cause, such as cerebral venous sinus thrombosis [2], the ingestion of tetracycline antibiotics, recombinant growth hormone, or lithium [3]. IIH/PTC was first described in 1937 by Walter Dandy in a case series of 22 patients, and the diagnostic criteria initially described continue to be used in contemporary studies [4,5]. This criterion was revised in 2013 and has been widely cited in the literature since [6]. The modified Dandy criteria currently require several conditions to be met for diagnosis: (1) a lumbar puncture must be performed and demonstrate elevated CSF pressure, greater than 25 cm H_2_O in adults or 28 cm H_2_O in children in the pediatric population, in the presence of symptoms suggestive of increased ICP such as headache, diplopia, pulsatile tinnitus, transient visual obscuration (TVO), or vision loss, 2) there must be no evidence of ventriculomegaly or a cerebral space-occupying lesion on radiological imaging, 3) optic disc edema must be present, 4) there should be no focal neurological deficits except for a possible abducens nerve palsy, and 5) supporting radiological findings may include an empty sella, dilated or tortuous optic nerve sheaths, a flattened lamina cribrosa, and cerebral venous sinus stenosis in the absence of thrombosis [6].

Previous studies on IIH epidemiology estimate the disease prevalence to be 0.03-1.7 cases per 100,000 people, depending on geographical location [7]. Risk factors for IIH include female sex, childbearing age, and obesity [7]. This disorder presents clinically with symptoms of a space-occupying lesion with headaches, pulsatile tinnitus, TVO, and optic disc edema with visual loss [1]. If IIH/PTC is left untreated, the primary morbidity is persistent vision loss, affecting 10% of patients [8].

While elevated ICP is required for the diagnosis of IIH/PTC, previous studies have not shown a linear association between elevated ICP at the time of diagnosis and visual impairment [9,10]. Therefore, the authors hypothesized that the hydrodynamics of optic disc edema may be a confounding variable that leads to vision loss in some but not all IIH/PTC patients. Translaminar pressure difference (TLPD) is a variable representing the net difference of oppositional intracranial and intraocular pressure (IOP) across the lamina cribrosa [11]. The authors hypothesized that TLPD would be useful in identifying patients with visual complaints secondary to IIH.

TLPD, defined as ICP minus IOP across the lamina cribrosa, has been implicated in optic nerve head biomechanics and glaucomatous injury, suggesting that the balance of pressures may influence vision outcomes, not pressure levels alone. Prior work has characterized the pressure gradient concept and its relevance to optic disc disease, largely in glaucoma populations. Building on this, we hypothesized that TLPD would be associated with specific visual complaints in IIH. To explore potential positional effects on pressure measurements, we compared associations in patients who underwent lumbar puncture in seated versus lateral decubitus positions.

## Materials and methods

Study design, setting, and participants

The study was conducted per all applicable national and institutional regulations. It was reviewed and approved by the Institutional Review Board of the affiliated medical college (IRB# HS201023-E). A single-center, retrospective, cross-sectional study was conducted in an outpatient neurology practice in the southeastern United States. Based on the ICD-10 diagnostic codes, all patients with IIH or PTC as the diagnosis between 2009 and 2020 were selected from the referring practice’s electronic health record. Initially, 408 patients were identified.

Patients with complete medical records meeting the diagnostic criteria for IIH/PTC as defined by the Friedman 2013 paper were initially included in the study. These criteria include 1) papilledema, 2) normal neurologic examination, 3) normal radiographic imaging without evidence of hydrocephalus, mass, or structural lesion and no abnormal meningeal enhancement on MRI, 4) normal CSF composition, and 5) elevated lumbar puncture opening pressure (>25 cm H_2_O) when lumbar puncture was performed in the supine position. Patients were excluded if they were under the age of 18, had a diagnosis of an active malignancy regardless of primary location, were currently pregnant, or had a secondary source of IIH identified. A total of 106 patients' data were included in the final statistical analysis. This was an exploratory convenience sample of all eligible patients during the study period. With n=106, the study was powered to detect medium to large effects; accordingly, we reported effect sizes alongside p-values and interpreted results cautiously for rare outcomes.

Measures

The following demographic variables were recorded from the patients’ charts at the time of diagnosis: age, sex, race (white versus racial minority), insurance coverage (full private, full governmental, combination of private and governmental insurance, none), body mass index (BMI), and smoking history (current or former smoking is considered positive). These (binary) outcome variables include clinical variables such as hypertension (HTN) diagnosis, headaches, tinnitus, dizziness, vision loss, blurred vision, TVO, or any vision problem (including vision loss, blurred vision, and or TVO). Visual complaints were predefined as any of the following recorded at diagnosis: blurred vision, TVO, or vision loss.

Intracranial and ophthalmologic pressure measurements were recorded for each patient. ICP is the opening pressure measured during a lumbar puncture and is expressed in millimeters of mercury (mmHg). Lumbar punctures were either performed in a seated or lateral decubitus position. If multiple ICP readings were recorded in the chart, the measurement from the time of diagnosis was included for statistical analysis. IOP was obtained from an ophthalmology consult note included in the patient’s electronic health record. This measurement is conceptualized as the eye fluid pressure measured in the standard fashion with tonometry. TLPD, which represents the opposing hydrostatic forces at the lamina cribrosa, was calculated by subtracting IOP from ICP measurements. All ICP values were analyzed in millimeters of mercury. When the opening pressure was recorded in centimeters of water, values were converted to millimeters of mercury by dividing by 1.36 before calculating TLPD. TLPD was calculated as ICP minus IOP, both in millimeters of mercury. If documented, the method of tonometry and timing relative to neurologic evaluation were abstracted from the ophthalmology note. Where method or timing was not recorded, IOP was treated as measured clinically per usual care. We acknowledge this heterogeneity as a limitation.

Analysis plan

Prior to conducting main analyses, demographic, health history, and pressure variables were examined for group differences (i.e., between patients undergoing lumbar puncture in a seated versus lateral decubitus position) using chi-squared (χ^2^) tests of independence for categorical variables and independent t-tests for continuous variables. We corrected analyses with Fisher’s exact test for 2x2 χ^2^ tests with cells of <5 values and Welch’s t-test for any violations of homogeneity of variance. We report effect sizes in-text for all significant associations and provide concise synthesis to avoid duplication. Findings in seated-group subcells with very small counts are presented descriptively.

For the main analyses, associations between study variables (HTN, smoking history, headache, tinnitus, dizziness, age, BMI, ICP, IOP, TLPD) and visual complaints, including vision loss, blurred vision, TVOs, or any combination of the three, were evaluated. Analyses were split between two patient groups based on position during lumbar puncture (seated versus lateral decubitus) due to considerable group differences, primarily in BMI and ICP, detailed in the results section.

The level of statistical significance (α) was set to 0.05 (two-tailed for preliminary descriptive analyses, one-tailed for main analyses). Additionally, for all analyses, we interpreted findings that met Ferguson’s recommended minimum effect size for what may be considered a “practically” significant effect (d≥0.41; φc≥0.20; OR≥2.0 or ≤0.5) [12]. The Statistical Package for the Social Sciences (SPSS) Version 25 (IBM Corp., Armonk, NY) was used for all statistical analyses. Analyses used complete-case data for each outcome. Denominators are reported in the tables when they vary. No imputation was performed. Because BMI and ICP differed by lumbar puncture position at baseline, we stratified analyses by position and report effect sizes. Given outcome frequencies and cell sizes, multivariable modeling was underpowered for several endpoints; we therefore treat findings as hypothesis-generating.

## Results

Full sample characteristics

Among 106 participants, 46 underwent lumbar puncture in a seated position and 60 in lateral decubitus. Most participants were female, 93 of 106 (87.7%), and White, 70 of 106 (66.0%), with a mean age of 36.8 years and a standard deviation of 12.5. Baseline comorbidities included HTN (37.1%), diabetes (13.5%), and tobacco use (42.9%). Headache was present in 88.5%, migraine in 32.7%, tinnitus in 47.6%, and dizziness in 36.2%. Visual symptoms at presentation were common: vision loss (12.3%), blurred vision (30.5%), TVO (36.5%), and any vision complaint (61.3%).

Lumbar puncture position comparisons

Groups were similar across most demographics and medical markers (Table 1). The seated group had a higher BMI mean of 43.87 (SD 11.64) than the lateral decubitus group mean of 36.56 (SD 7.76). In contrast, the lateral decubitus group showed higher ICP (mean 36.54, SD 7.96 versus 29.20, SD 3.39), higher TLPD (mean 36.73, SD 12.16 versus 21.00, SD 10.57), and a higher base rate of vision loss (18.3% versus 4.3%). No other between-group differences were observed.

**Table 1:** Demographic and clinical characteristics of the study sample of participants with pseudotumor cerebri (n=106), with between-group comparisons for lumbar puncture position *The p-value reflects Fisher's exact test. ^†^The p-value reflects Welch’s t-test. BMI, body mass index; ICP, intracranial pressure; IOP, intraocular pressure; LD, lateral decubitus; OR, odds ratio; TLP, translaminar pressure; TVO, transient visual obscuration

Variable	Lumbar puncture position		Comparison
	Seated (n=46)	LD (n=60)	
Age, M (SD)	38.30 (11.99)	35.73 (12.95)	t=1.05, p=0.298, d=0.21
Sex, n (%)	-	-	χ^2^=0.66, p=0.417, OR=1.62
Female	39 (84.8)	54 (90.0)	-
Male	7 (15.2)	6 (10.0)	-
Race, n (%)	-	-	χ^2^=0.33, p=0.569, OR=0.79
Racial minority	17 (37.0)	19 (31.7)	-
White	29 (63.0)	41 (68.3)	-
Insurance, n (%)	-	-	χ^2^=3.78, p=0.286, φ_c_=0.189
Private	34 (73.9)	39 (65.0)	-
Government	9 (19.6)	10 (16.7)	-
Mixed	2 (4.3)	4 (6.7)	-
None	1 (2.2)	7 (11.7)	-
Hypertension, n (%)	17 (37.0)	22 (37.3)	χ^2^<0.01, p=0.972, OR=1.01
Smoking history, n (%)	19 (41.3)	26 (44.1)	χ^2^=0.08, p=0.776, OR=1.12
Headache, n (%)	41 (91.1)	51 (86.4)	χ^2^=0.55, p=0.547*, OR=0.62
Tinnitus, n (%)	22 (48.9)	28 (46.7)	χ^2^=0.05, p=0.821, OR=0.92
Dizziness, n (%)	15 (32.6)	23 (39.0)	χ^2^=0.46, p=0.500, OR=1.32
Vision loss, n (%)	2 (4.3)	11 (18.3)	χ^2^=4.73, p=0.037*, OR=4.94
Blurred vision, n (%)	13 (28.3)	19 (32.2)	χ^2^=0.19, p=0.663, OR=1.21
TVO, n (%)	20 (44.4)	18 (30.5)	χ^2^=2.14, p=0.144, OR=0.55
Any vision problem, n (%)	31 (67.4)	34 (56.7)	χ^2^=1.26, p=0.261, OR=0.63
BMI, M (SD)	43.87 (11.64)	36.56 (7.76)	t=3.85, p<0.001 d=0.74
ICP, M (SD)	29.20 (3.39)	36.54 (7.96)	t=5.86, p<0.001^†^, d=1.20
IOP, M (SD)	37.36 (8.55)	35.18 (9.24)	t=0.95, p=0.347, d=0.24
TLP, M (SD)	21.00 (10.57)	36.73 (12.16)	t=-5.34, p<0.001 d=1.38

Demographic and vision characteristics in the seated PTC sample

Tables 2, 3 display categorical and continuous variables, respectively, concerning vision problems, split by lumbar puncture position. For seated participants, reported history of tinnitus was associated with 7.29 times higher odds of TVO (75.0% vs. 29.2%, p=0.001). No other associations with vision symptoms were observed among the study variables of interest.

**Table 2:** Categorical variables in relation to vision problems in participants with pseudotumor cerebri, stratified by lumbar puncture position. *The p-value reflects Fisher's exact test. Numbers and percentages are anchored within-columns (e.g., the base rate of HTN among those with LD lumbar puncture position and vision loss is 45.5%). HTN, hypertension; LD, lateral decubitus; OR, odds ratio

Position	Vision loss					Blurred vision					Transient visual obscuration					Any vision problem				
Variable	No, n (%)	Yes, n (%)	χ^2^	p	OR	No, n (%)	Yes, n (%)	χ^2^	p	OR	No, n (%)	Yes, n (%)	χ^2^	p	OR	No, n (%)	Yes, n (%)	χ^2^	p	OR
Seated																				
HTN	15 (34.1)	2 (100)	-	-	-	10 (30.3)	7 (53.8)	2.22	0.068	2.68	10 (40.0)	6 (30.0)	0.49	0.757	0.64	5 (33.3)	12 (38.7)	0.13	0.362	1.26
Smoking	17 (38.6)	2 (100)	-	-	-	14 (42.4)	5 (38.5)	0.06	0.597	0.85	9 (36.0)	9 (45.0)	0.38	0.270	1.46	5 (33.3)	14 (45.2)	0.58	0.223	1.65
Headache	39 (90.7)	2 (100)	-	-	-	30 (93.8)	11 (84.6)	0.95	0.716*	0.37	21 (87.5)	19 (95.0)	0.74	0.307*	2.71	13 (92.9)	28 (90.3)	0.08	0.500*	0.72
Tinnitus	22 (51.2)	0 (0.0)	-	-	-	18 (56.3)	4 (30.8)	2.40	0.905	0.35	7 (29.2)	15 (75.0)	9.17	0.001	7.29	4 (28.6)	18 (58.1)	3.36	0.054*	3.46
Dizziness	14 (31.8)	1 (50.0)	-	-	-	10 (30.3)	5 (38.5)	0.28	0.298	1.44	10 (40.0)	5 (25.0)	1.13	0.855	0.50	6 (40.0)	9 (29.0)	0.55	0.771	0.61
LD																				
HTN	17 (35.4)	6 (45.5)	0.39	0.268	1.52	12 (30.0)	10 (55.6)	3.44	0.032	2.92	16 (39.0)	6 (33.3)	0.17	0.661	0.78	8 (30.8)	14 (42.4)	0.85	0.179	1.66
Smoking	20 (41.7)	6 (54.5)	0.60	0.219	1.68	15 (37.5)	11 (61.1)	2.80	0.047	2.62	16 (39.0)	10 (55.6)	1.39	0.120	1.95	7 (26.9)	19 (57.6)	5.54	0.010	3.68
Headache	41 (85.4)	10 (90.9)	0.23	0.500*	1.71	32 (82.1)	18 (94.7)	1.73	0.126*	3.94	34 (85.0)	16 (88.9)	0.16	0.500*	1.41	20 (80.0)	31 (91.2)	1.54	0.133*	2.58
Tinnitus	23 (46.9)	5 (45.5)	0.01	0.535	0.94	18 (45.0)	10 (52.6)	0.30	0.292	1.36	17 (41.5)	10 (55.6)	1.00	0.159	1.77	10 (38.5)	18 (52.9)	1.24	0.133	1.80
Dizziness	16 (33.3)	7 (63.6)	3.46	0.032	3.50	14 (35.0)	9 (50.0)	1.17	0.140	1.86	14 (34.1)	9 (50.0)	1.32	0.125	1.93	6 (23.1)	17 (51.5)	4.94	0.013	3.54

**Table 3:** Continuous variables in relation to vision problems in participants with pseudotumor cerebri, stratified by lumbar puncture position. Means and SDs are anchored within columns (e.g., the mean age among those with LD lumbar puncture position and with vision loss=38.1±15.6). *The p-value reflects Welch’s t-test. BMI, body mass index; ICP, intracranial pressure; IOP, intraocular pressure; LD, lateral decubitus; TLP, translaminar pressure

Position	Vision Loss				Blurred vision				Transient visual obscuration				Any vision problem			
Variable	No, M (SD)	Yes, M (SD)	t	p	No, M (SD)	Yes, M (SD)	t	p	No, M (SD)	Yes, M (SD)	t	p	No, M (SD)	Yes, M (SD)	t	p
Seated																
Age	38.2 (12.2)	41.5 (3.5)	-	-	37.5 (10.1)	40.4 (16.1)	-0.60*	0.278	39.5 (12.0)	36.8 (12.4)	0.76	0.453	40.3 (10.1)	37.3 (12.8)	0.80	0.784
BMI	43.7 (11.7)	48.2 (12.5)	-	-	43.4 (12.4)	45.2 (9.8)	-0.47	0.320	45.5 (14.2)	41.2 (6.8)	1.25	0.217	46.9 (16.8)	42.4 (8.1)	1.24	0.888
ICP	29.2 (3.4)	28.5 (3.5)	-	-	29.7 (3.6)	27.9 (2.4)	1.63	0.944	29.2 (3.7)	29.3 (3.0)	-0.06	0.954	29.9 (4.3)	28.8 (2.9)	0.90*	0.810
IOP	36.6 (8.4)	47.5 (2.1)	-	-	36.9 (8.3)	38.7 (9.7)	-0.48	0.681	37.8 (7.8)	36.0 (9.8)	0.52	0.611	38.8 (7.2)	36.4 (9.4)	0.72	0.239
TLP	21.9 (10.4)	9.5 (4.9)	-	-	22.3 (10.3)	17.0 (11.1)	1.16	0.872	21.0 (10.3)	22.4 (10.9)	-0.33	0.744	20.8 (10.9)	21.1 (10.7)	-0.07	0.472
LD																
Age	35.2 (12.4)	38.1 (15.6)	-0.67	0.255	35.5 (12.9)	35.7 (13.5)	-0.07	0.472	35.6 (13.3)	35.7 (12.9)	-0.01	0.993	36.5 (14.7)	35.2 (11.6)	0.40	0.654
BMI	36.8 (7.9)	35.3 (7.2)	0.57	0.715	37.4 (8.1)	35.0 (7.1)	1.09	0.859	37.3 (8.1)	35.2 (7.1)	0.93	0.355	37.7 (8.6)	35.7 (7.0)	0.98	0.835
ICP	36.2 (7.2)	37.9 (11.0)	-0.48*	0.320	35.6 (7.0)	38.6 (9.8)	-1.22*	0.118	37.3 (8.9)	34.3 (4.7)	1.67*	0.100	34.6 (6.0)	38.0 (9.0)	-1.74*	0.044
IOP	36.1 (9.2)	26.3 (3.1)	1.80	0.041	35.7 (10.1)	33.8 (7.0)	0.51	0.309	35.6 (10.6)	34.4 (6.1)	0.36	0.725	36.1 (11.4)	34.2 (6.5)	0.59	0.279
TLP	36.6 (12.7)	38.3 (6.1)	-0.24	0.408	34.3 (11.0)	44.0 (14.1)	-2.01	0.027	38.5 (13.0)	33.3 (10.0)	1.16	0.255	35.6 (11.2)	37.9 (13.3)	-0.55	0.294

Associations with visual complaints

Analyses of continuous measures identified three outcome-specific associations. Within the lateral decubitus subgroup, blurred vision was associated with higher TLPD (mean 44.0, SD 14.1 versus 34.3, SD 11.0, p = 0.03, Cohen's d = 0.82). Vision loss was associated with lower IOP (mean 26.3, SD 3.1 versus 36.1, SD 9.2, p = 0.04, Cohen's d = 1.09). Reporting any vision complaint was associated with higher ICP (mean 38.0, SD 9.0 versus 34.6, SD 6.0, p = 0.04, Cohen's d = 0.44). No other statistically significant differences were observed.

## Discussion

IIH and its impact on visual health present a dynamic association that warrants exploration, especially given the paucity of specific risk associations. The associations between IIH and potential risk factors that can precipitate the onset of vision pathologies may allow for further prioritization of treatments and highlight pertinent concerns and findings. Results from this study support historically described risk factors for IIH [1]. This study also highlights the new association between TLPD and blurry vision.

This study's findings reveal a notable prevalence of visual complaints within this sample, ranging from 12% to 37%, with 61% of participants reporting at least one form of visual complaint. Notably, factors such as a higher BMI, lower ICP, and lower TLPD were more prevalent in the seated group compared to the lateral decubitus group.

In seated patients, tinnitus was associated with TVO, but this may reflect symptom clustering rather than a mechanistic link. This requires confirmation in larger prospective cohorts. Conversely, the lateral decubitus group exhibited a more diverse set of associations. Dizziness, lower IOP, smoking history, and higher TLPD correlated with vision loss, blurred vision, and any other vision complaints. The role of TLPD has been recently correlated with primary open-angle glaucoma [13]. In clinical practice, documenting both IOP and ICP allows calculation of TLPD, which in this cohort was tracked with blurred vision in lateral decubitus measurements. While exploratory, these data suggest that TLPD may help identify patients at risk for blurred vision and could inform closer ophthalmic follow-up.

This study has several limitations. First, the cross-sectional, single-center design limits causal inference and may reduce generalizability beyond our regional referral population. Second, ICP and IOPs were obtained as part of routine care rather than a standardized protocol. Tonometry method and timing were variably documented, lumbar puncture position differed across patients, and ICP recorded in centimeters of water was converted to millimeters of mercury before calculating the TLPD. Second, outcomes were defined from clinical documentation of visual complaints rather than uniform objective ophthalmic testing such as perimetry or optical coherence tomography, which may introduce misclassification. Fourth, some subgroups, particularly those with vision loss, were small. As a result, estimates were imprecise, multivariable adjustment was limited for several endpoints, and findings should be interpreted as hypothesis-generating despite reporting effect sizes.

## Conclusions

IIH shows outcome-specific relationships between visual symptoms and pressure measures that span the brain and the eye. After stratifying by lumbar puncture position, higher TLPD correlated with blurred vision in the lateral decubitus group, and lower IOP correlated with vision loss. Any vision complaint is tracked with higher ICP. Taken together, these findings support potentially routine documentation of IOP alongside ICP so that the TLPD can be calculated and used to inform risk assessment and follow-up. Given the single-center, cross-sectional design, heterogeneity in IOP measurement, and small subgroups for some outcomes, these results should be considered hypothesis-generating. Prospective studies with standardized pressure measurements and predefined ophthalmic endpoints are needed to validate thresholds and define how best to integrate these markers into clinical care.
